# Physical Activity Is Associated With Macular Thickness: A Multi-Cohort Observational Study

**DOI:** 10.1167/iovs.64.3.11

**Published:** 2023-03-03

**Authors:** Ella C. Berry, Henry N. Marshall, Sean Mullany, Santiago Diaz Torres, Joshua Schmidt, Daniel Thomson, Lachlan S. W. Knight, Georgina L. Hollitt, Ayub Qassim, Bronwyn Ridge, Angela Schulz, Mark M. Hassall, Thi Thi Nguyen, Stewart Lake, Richard A. Mills, Ashish Agar, Anna Galanopoulos, John Landers, Paul R. Healey, Stuart L. Graham, Alex W. Hewitt, Stuart MacGregor, Robert J. Casson, Owen M. Siggs, Jamie E. Craig

**Affiliations:** 1 Department of Ophthalmology, Flinders Health and Medical Research Institute, Flinders University, Adelaide, South Australia, Australia; 2 QIMR Berghofer Medical Research Institute, Herston, Queensland, Australia; 3 Faculty of Health and Medical Sciences, Macquarie University, Sydney, Australia; 4 Department of Ophthalmology, University of New South Wales, Sydney, New South Wales, Australia; 5 Discipline of Ophthalmology and Visual Sciences, University of Adelaide, Adelaide, South Australia, Australia; 6 Centre for Vision Research, University of Sydney, Sydney, New South Wales, Australia; 7 Menzies Institute for Medical Research, University of Tasmania, Hobart, Tasmania, Australia; 8 Garvan Institute of Medical Research, Sydney, New South Wales, Australia

**Keywords:** macular, physical activity, optical coherence tomography, aging, exercise

## Abstract

**Purpose:**

To assess the association between physical activity and spectral-domain optical coherence tomography (SD-OCT)–measured rates of macular thinning in an adult population with primary open-angle glaucoma.

**Methods:**

The correlation between accelerometer-measured physical activity and rates of macular ganglion cell–inner plexiform layer (GCIPL) thinning was measured in 735 eyes from 388 participants of the Progression Risk of Glaucoma: RElevant SNPs with Significant Association (PROGRESSA) study. The association between accelerometer-measured physical activity and cross-sectional SD-OCT macular thickness was then assessed in 8862 eyes from 6152 participants available for analysis in the UK Biobank who had SD-OCT, ophthalmic, comorbidity, and demographic data.

**Results:**

Greater physical activity was associated with slower rates of macular GCIPL thinning in the PROGRESSA study (beta = 0.07 µm/y/SD; 95% confidence interval [CI], 0.03–0.13; *P* = 0.003) after adjustment for ophthalmic, demographic and systemic predictors of macular thinning. This association persisted in subanalyses of participants characterized as glaucoma suspects (beta = 0.09 µm/y/SD; 95% CI, 0.03–0.15; *P* = 0.005). Participants in the upper tertile (greater than 10,524 steps/d) exhibited a 0.22-µm/y slower rate of macular GCIPL thinning than participants in the lower tertile (fewer than 6925 steps/d): −0.40 ± 0.46 µm/y versus −0.62 ± 0.55 µm/y (*P* = 0.003). Both time spent doing moderate/vigorous activity and mean daily active calories were positively correlated with rate of macular GCIPL thinning (moderate/vigorous activity: beta = 0.06 µm/y/SD; 95% CI, 0.01–0.105; *P* = 0.018; active calories: beta = 0.06 µm/y/SD; 95% CI, 0.006–0.114; *P* = 0.032). Analysis among 8862 eyes from the UK Biobank revealed a positive association between physical activity and cross-sectional total macular thickness (beta = 0.8 µm/SD; 95% CI, 0.47–1.14; *P* < 0.001).

**Conclusions:**

These results highlight the potential neuroprotective benefits of exercise on the human retina.

Physical activity has been proposed to be protective against multiple ophthalmic diseases of aging. Epidemiological studies have conferred correlations between daily exercise and lower rates of open-angle glaucoma, age-related macular degeneration, and diabetic retinopathy.^1^^–7^ Furthermore, exercise has been associated with improved patient outcomes in these diseases.^8^^–^^10^ Potential mechanisms implicated include enhanced expression of brain-derived neurotrophic factor, improved retinal perfusion, and decreased intraocular pressure (IOP).^11^^–^^13^

No large-scale study to date has correlated exercise with spectral-domain optical coherence tomography (SD-OCT)–derived metrics of retinal thickness. SD-OCT offers an objective and reproducible measurement of neuroretinal thickness.^14^ Correlating exercise with objectively measured markers of neuroretinal degeneration may facilitate further studies evaluating the neuroprotective benefits of exercise in ophthalmic disease–specific cohorts. This study assessed the relationship between physical activity and macular thinning. In the primary analysis, it investigated the correlation between accelerometer–measured physical activity (measured in daily step count) and rate of longitudinal macular ganglion cell–inner plexiform layer (GCIPL) thinning in a well-characterized observational cohort of adults with early primary open-angle glaucoma. A secondary analysis was then performed correlating accelerometer-measured physical activity with cross-sectional SD-OCT–derived macular thickness in a population-based cohort of adult participants from the UK Biobank.

## Methods

### Study Overview

Accelerometer-measured physical activity was correlated with the rate of SD-OCT macular GCIPL thinning in the Progression Risk of Glaucoma: RElevant SNPs with Significant Association (PROGRESSA) study. We then performed a second analysis correlating accelerometer-measured physical activity with macular thickness in 6152 subjects from the UK Biobank. The PROGRESSA and the UK Biobank studies were approved by local research ethics boards. Informed consent was obtained from all participants, and all research adhered to the tenets of the Declaration of Helsinki.

### PROGRESSA Study Cohort Description

The PROGRESSA study is an ongoing, longitudinal, multisite, cohort study of suspect and early manifest primary open-angle glaucoma patients in Australia. Participants are prospectively recruited following referral from primary-care providers to ophthalmologists and are reviewed every 6 months for a minimum of 5 years. Glaucoma management is at the discretion of the treating clinician following standard clinical practice. Inclusion criteria for PROGRESSA require the presence of an optic disc suspicious of glaucoma, ocular hypertension, or a mild glaucomatous visual field defect with a mean deviation better than −6.0 decibels on two reliable visual fields. Participants with secondary forms of glaucoma, narrow iridocorneal angle or previous peripheral iridotomy, or a previous diagnosis of pseudo-exfoliation syndrome or pigment dispersion syndrome at enrollment were excluded. Participants with myopia of >10 diopters and retinal or neurological causes of visual field loss are excluded from enrollment in the study.^15^ Other inclusion criteria for this particular study included willingness to wear a wrist-worn accelerometer for 7 days. Participants were able to remove the device and cease involvement in the study at any time. Participants were excluded if they had been hospitalized or undergone surgery in the preceding 6 weeks. Participants in this specific study had been enrolled into the PROGRESSA study between May 2012 and March 2022.

Five hundred and twelve participants from the PROGRESSA study were consecutively invited to participate in this study during routine PROGRESSA clinics between January 2021 and March 2022. Participants who accepted the invitation wore a Fitbit Inspire 2 (Fitbit, San Francisco, CA, USA) on their non-dominant wrist for a continuous 7-day period during all waking hours (including when bathing or swimming). Fitbit Inspire 2 quantifies daily step count, total energy expenditure, and time spent in moderate or vigorous intensity exercise.^16^ Mean daily number of steps during the study period was the metric of choice because this afforded an objective measure that was not confounded by basal heart rate or arrhythmias. Moderate physical activity is defined as time spent with heart rate between 60% and 84% of maximum heart rate, and vigorous physical activity is defined as time spent with heart rate greater than 84% of maximum heart rate. Individuals who did not complete 7 days wearing the device were excluded from analysis. The 7-day study monitoring period commenced on the day following the ophthalmic review during which they were recruited and fitted with the device. All participants were blinded to their daily step counts. The decision to use the non-dominant hand was as per Fitbit Inspire 2 device instructions.

Retrospective rates of SD-OCT macular GCIPL thinning were calculated using CIRRUS HD-OCT 11.0 software (Carl Zeiss Meditec, Dublin, CA, USA). Participants enrolled in the PROGRESSA study undergo 6-monthly 512 × 128-mm macular cube and 200 × 200-mm optic nerve head imaging at enrollment and every 6 months during monitoring. Rates of macular GCIPL thinning were calculated from enrollment into PROGRESSA up until and including the date of the Fitbit Inspire 2 device fitting, using CIRRUS HD-OCT Trend Analysis software. The macular GCIPL thickness was defined as the average thickness of retinal tissue between the peripapillary retinal nerve fiber layer (RNFL) and the inner plexiform layer, as per the CIRRUS HD-OCT segmentation protocol. Per the CIRRUS HD-OCT Trend Analysis protocol, the rate of macular GCIPL is calculated using the first two baseline scans and the most recent six scans (at maximum), with exclusion of any intervening scans. SD-OCT scans were performed using CIRRUS FastTrac eye-tracking technology. A single investigator (HNM) evaluated all SD-OCT scans for image quality. Scans with a signal strength of less than 6, a significant acquisition artifact, or a focal pathological artifact (e.g., macular hole, macular edema) were excluded. For secondary analysis using longitudinal peripapillary RNFL thickness data, rates of peripapillary RNFL thinning were similarly calculated from enrollment up to and including data for the Fitbit Inspire 2 device fitting using CIRRUS HD-OCT Trend Analysis of the segmented peripapillary RNFL from the 200 × 200-mm optic nerve head scan.

The PROGRESSA study protocol stipulates that the following ophthalmic measurements are recorded by the reviewing clinician at enrollment and subsequent reviews: best-corrected central visual acuity, IOP (Goldmann applanation tonometry), ultrasound central corneal pachymetry, and vertical cup-to-disc ratio. Medical histories were acquired verbally using a standardized participant questionnaire that addressed the following cardiovascular disease risk factors: hypertension, diabetes, myocardial infarction, self-reported medications (antihypertensives, lipid-lowering therapy, or antiplatelet agents), and current or past smoking status. Systolic and diastolic blood pressures were measured using an automated sphygmomanometer. Socioeconomic status was estimated using publicly available postcode-derived deciles of socioeconomic disadvantage.^17^ Participants were additionally characterized as either glaucoma suspect or early manifest glaucoma by a fellowship-trained subspecialist ophthalmologist (JEC) using modified Hodapp–Parrish–Anderson criteria on consecutive reliable 24-2 Humphrey visual field assessments (Humphrey Field Analyzer; Carl Zeiss Meditec).^18^^–^^20^

### UK Biobank Cohort Description

A secondary analysis was performed by correlating physical activity with cross-sectional retinal macular thickness in the UK Biobank. The UK Biobank is a national cohort study of participants 40 to 69 years of age in the United Kingdom. Non-mydriatic macular SD-OCT imaging was conducted using the Topcon 3D OCT-1000 Mark II device (three-dimensional 6 × 6-mm^2^ volume scans; Topcon, Tokyo, Japan) between 2009 and 2010.^21^ Macular thickness was estimated as the mean distance between the inner limiting membrane (ILM) and the retinal pigment epithelium (RPE) across the 128 B-scans that comprise the 6 × 6-mm^2^ macular volume cube. The ILM and the RPE were segmented using Version 1.6.1.1 of the Topcon Advanced Boundary Segmentation algorithm.^21^ Eyes with a low signal-to-noise ratio or a poor segmentation were excluded from analysis.^21^

Physical activity in the UK Biobank was estimated using accelerometer-measured physical activity in 103,705 participants between February 2013 and December 2015.^22^ Participants were invited to wear a triaxial accelerometer on their dominant wrist continuously for 7 days (Axivity AX3; Axivity, Newcastle Upon Tyne, UK). These devices captured three-dimensional acceleration data at 100 Hz with a dynamic range of ±8*g*. Participants’ data were excluded if they wore the accelerometer for less than 72 hours or did not have a data point in each 1-hour period of each 24-hour cycle of each day of the study period.^22^ Because such physical activity data may be confounded by differences in local gravity, the physical activity data for each participant had to be adjusted for differences in local gravity. This adjustment (or calibration) was done by comparing measurements at time points when a participant was stationary to measurements when the previous participant who wore the same device was stationary. As per the methods described by Doherty and colleagues,^22^ participants were also excluded if they did not have enough stationary data to perform this calibration. A full description of the data collection, processing, and analysis can be found elsewhere.^22^

The following International Classification of Disease, Tenth Revision (ICD-10) diagnoses that are hypothesized to affect macular thickness were recorded: diabetes mellitus, hypertension, Alzheimer’s disease, and stroke. The following ophthalmic conditions, considered predictive of macular thickness, were also recorded: ICD-10 diagnosis of glaucoma, ICD-10 diagnosis of retinal disease, and per-eye IOP. Age and sex were also recorded. Details regarding the UK Biobank can be found elsewhere.^23^

### Statistical Analysis

Analysis of the association between physical activity (mean daily step count) and rate of macular GCIPL thinning in the PROGRESSA cohort was conducted using a stepwise approach to regression. An initial univariable mixed-effects regression analysis assessed the correlation between mean daily step count and rate of macular GCIPL thinning. A second model was then constructed accounting for age and gender, and then a third final model was constructed accounting for covariates selected a priori with biological plausibility or previously documented correlations with macular thinning. A random intercept per patient was included in all analyses to account for the inclusion of more than one eye per patient (lme4 package in R; R Foundation for Statistical Computing, Vienna, Austria). Covariates for the final analysis included demographic (age and gender), ocular (maximum recorded IOP, baseline macular GCIPL thickness), and cardiovascular traits (maximum recorded systolic blood pressure, presence of diabetes mellitus). A secondary analysis using the final model subanalyzed participants characterized as glaucoma suspects. For descriptive purposes, participants were stratified into tertiles of physical activity based on mean daily step counts during the 7-day study period.

Having demonstrated associations between daily step count and macular GCIPL thinning, we then assessed the correlation between daily step counts and rates of peripapillary RNFL thinning, and we assessed the correlation between alternative measures of physical activity with rates of macular GCIPL thinning (mean time spent doing moderate/vigorous activity per day, mean daily active calorie expenditure). All secondary analyses utilized the same covariates as in the primary analysis and were performed using mixed-effects linear regression analysis. The *P* value for statistical significance in the secondary analysis of alternative measures of physical activity was set at 0.025 (Bonferroni correction). Physical activity was also correlated with each covariate using univariable linear regression to enable discussion of potential mediating pathways of effect.

Similarly, regression analysis in the UK Biobank was conducted using a stepwise approach. Initial univariable analysis was followed by a second analysis adjusting for age and gender, before a final multivariable regression analysis adjusting for traits with biological or known associations with macular thickness. The following traits were selected for inclusion in the model: age, gender, eye (right vs. left), IOP, cardiovascular traits (hypertension and diabetes), and neurological traits (history of stroke, Alzheimer's disease, or Parkinson's disease). Prior to construction of the final multivariable model, the association of these variables with total macular thickness was tested using multivariable regression. Variable variables demonstrating *P* > 0.1 were excluded from the final analysis. The measure of physical activity (internally centered and scaled milligravity) was then included in the multivariable modeling. Secondary analysis excluded participants with known ophthalmic conditions.

The summary tabulated data present the mean ± SD for continuous variables and the number and prevalence (percent) for discrete variables in the PROGRESSA study cohort. All continuous variables were internally scaled to *z*-scores prior to model fitting. Beta coefficients with 95% confidence intervals (CIs) were used to represent the 1-SD increase for continuous variables on the macular thickness.

## Results

### Association of Physical Activity and Rate of Macular Thinning in PROGRESSA Cohort

**Figure 1.**
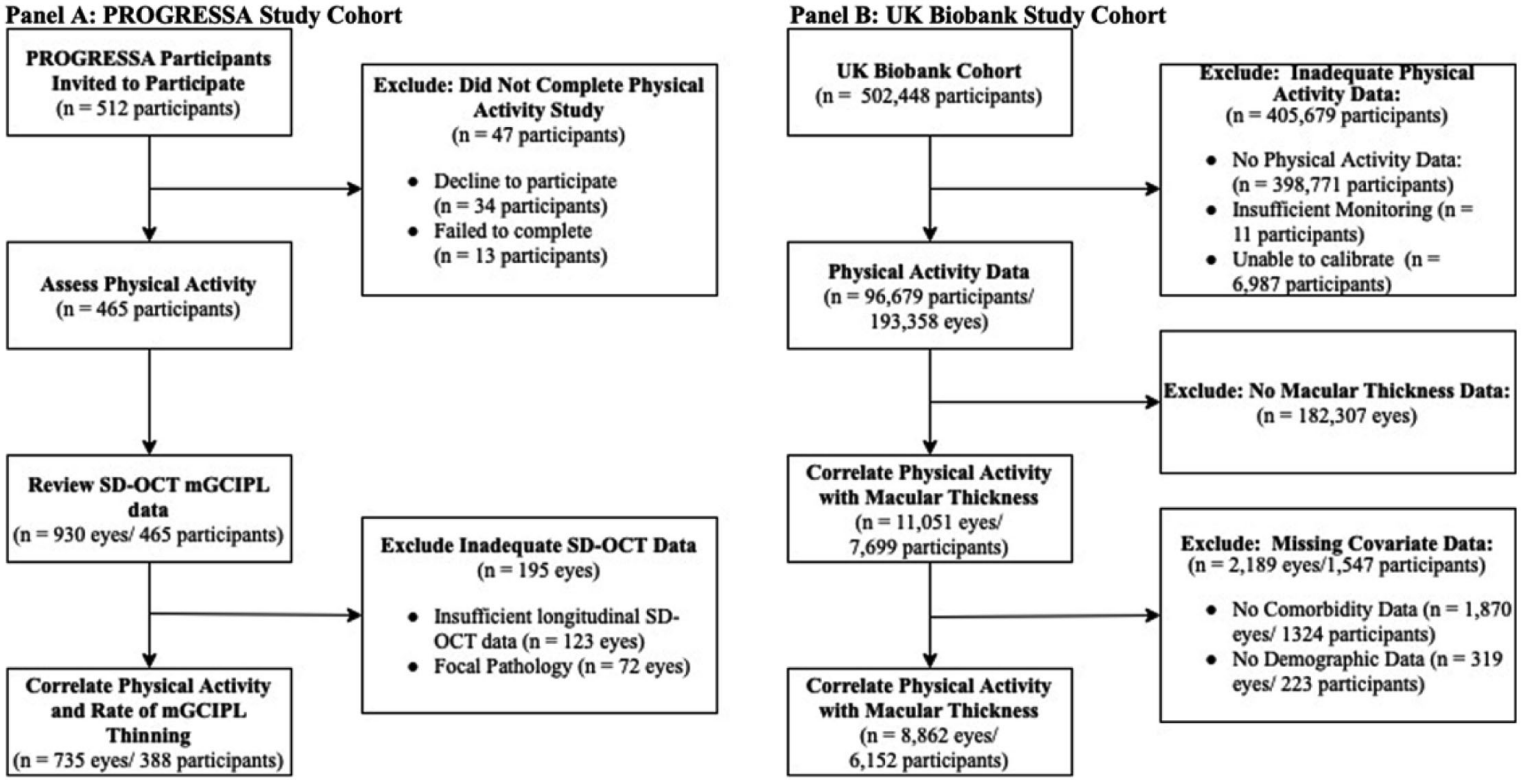
Study cohort schematic. (**A**) In this study, 465 participants in the PROGRESSA cohort successfully completed the 7-day study period. Following review of SD-OCT imaging, the rates of macular GCIPL thinning were correlated with recorded mean daily steps in 735 eyes from 388 participants. (**B**) In the secondary analysis, 96,679 participants of the UK Biobank successfully completed the physical activity study. Of the potential 193,358 eyes of this cohort, 11,051 eyes had available macular thickness data. A further 2189 eyes (*n* = 1547 participants) were then excluded due to unavailable comorbidity or demographic data, leaving 8862 eyes from 6152 participants for analysis.

Five hundred and twelve PROGRESSA participants were invited to wear a Fitbit Inspire 2 device. Of these participants, 34 declined and 13 failed to complete the 7-day study period. The mean daily step count was calculated for the 465 participants who completed the 7-day study period. The longitudinal CIRRUS SD-OCT macular GCIPL data for these 465 (*n* = 930 eyes) participants was reviewed. Of the 930 eyes, 807 had sufficient SD-OCT imaging to calculate the rate of macular GCIPL thinning as per the Zeiss CIRRUS HD-OCT protocol (minimum four scans), of which a further 72 eyes were excluded due to defects (focal pathology, segmentation defects), leaving 735 eyes with high-quality longitudinal macular GCIPL data (*n* = 388 participants) (Fig. 1A).

The mean duration of monitoring was 5.6 ± 2.3 years, and the median number of scans was eight (interquartile range [IQR], 7–8). The mean age of the study participants was 70.4 ± 8.8 years, 56.7% were female, the average maximum record IOP was 20.6 ± 5.5 mmHg, and the average rate of macular GCIPL thinning was −0.48 ± 0.57 µm/y. The mean daily step count was 9002 steps with a standard deviation of 4159 steps. Participants with greater than 10,524 steps/d were characterized as the upper tertile, and participants with fewer than 6925 steps/d were characterized as the lower tertile. (Supplementary Table S1).

Analysis of covariates of the rate of macular GCIPL thinning determined that age (*P* = 0.004), maximum recorded IOP (*P* < 0.001), and maximum systolic blood pressure (*P* = 0.032) were associated with faster rates of macular GCIPL thinning. Gender (*P* = 0.849) and baseline average thickness (*P* = 0.352) were not associated with rate of macular GCIPL thinning. Of these parameters, physical activity was negatively correlated with age (beta = −2.56 y/SD; 95% CI, −3.16 to −1.93; *R*^2^ = 0.08; *P* < 0.001) and with systolic blood pressure (beta = −3.95 mmHg/SD; 95% CI, 2.54–5.35; *R*^2^ = 0.04; *P* < 0.001).

**Figure 2.**
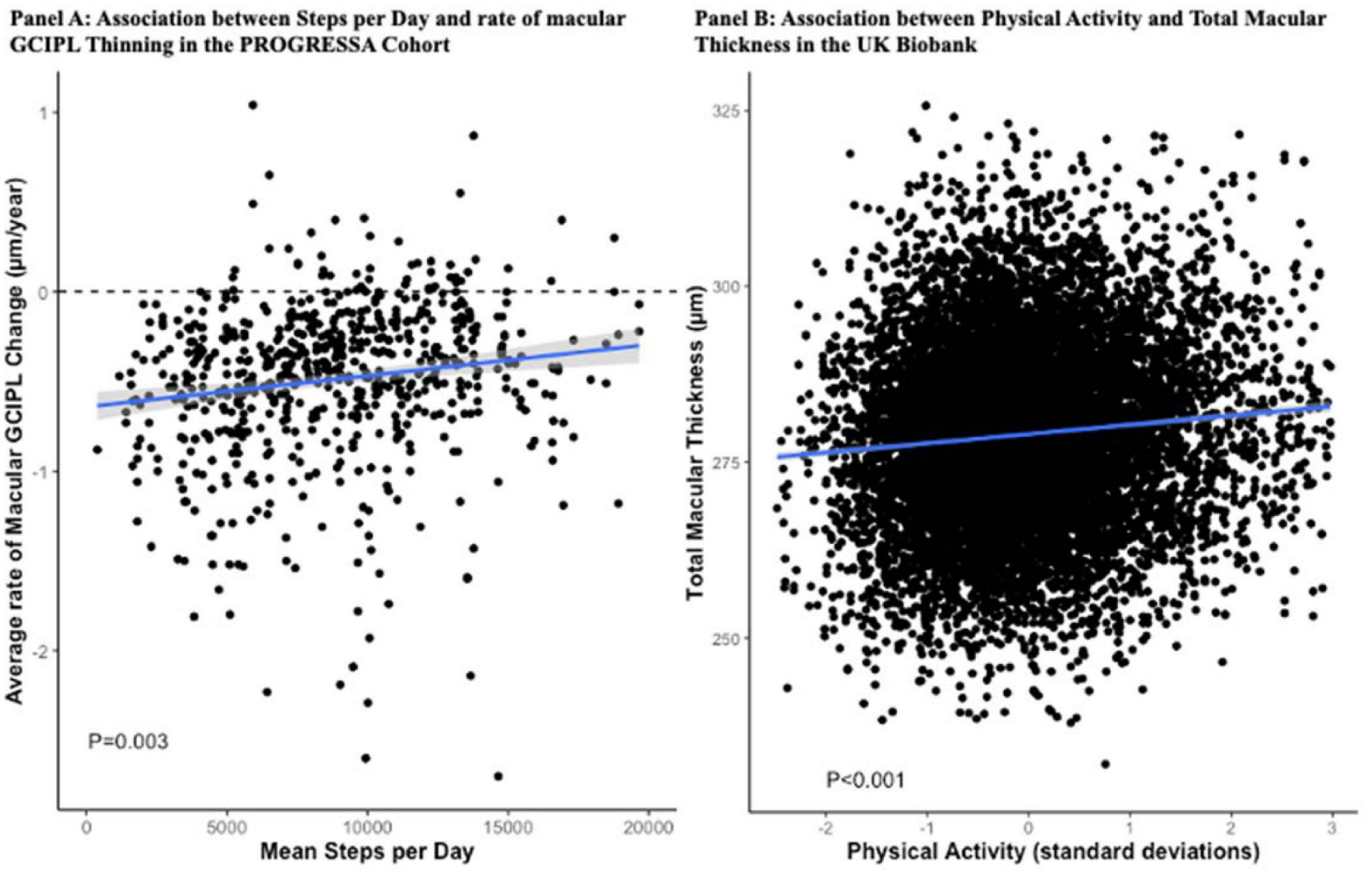
Correlation between mean daily step count and rate of macular thinning in the PROGRESSA cohort and physical activity and macular thickness in the UK Biobank cohort. (**A**) Scatterplot depicting the association between mean daily step count (*x*-axis) and rate of change in macular thickness (*y*-axis). The *blue line* indicates the linear regression between step count and rate of macular thinning with 95% confidence intervals (*gray shaded area*). The *dashed* *line* is representative of a rate of change of 0 µm/y. In the PROGRESSA cohort, a higher daily step count was correlated with a slower rate of macular GCIPL thinning. The *P* value was derived from multivariable analysis. (**B**) Scatterplot depicting the association between physical activity (*x*-axis) and total macular thickness in the UK Biobank (*y*-axis). The *blue* *line* indicates the linear regression between standardized physical activity and total macular thickness with 95% confidence intervals (*gray shaded area*). In the UK Biobank, greater physical activity was associated with greater total macular thickness after adjustment for age, gender, and associated ocular and systemic predictors of macular thickness. The *P* value was derived from multivariable analysis.

Physical activity was associated with the rate of macular GCIPL thinning in univariable analysis (beta = 0.09 µm/y/SD; 95% CI, 0.05, 0.12; *P* < 0.001), in subsequent analyses adjusting initially for age and gender (beta = 0.08 µm/y/SD; 95% CI, 0.04–0.13; *P* = 0.001), and in the final multivariable regression analysis incorporating additional ocular and cardiovascular traits (beta = 0.07 µm/y/SD; 95% CI, 0.03–0.13; *P* = 0.003) (Table, Fig. 2A). Participants in the upper tertile exhibited a 0.22-µm/y slower rate of macular GCIPL thinning than participants in the lower tertile (−0.40 ± 0.46 µm/y vs. −0.62 ± 0.55 µm/y). This association persisted in subanalyses of participants characterized as glaucoma suspects (multivariable beta = 0.09µm/y/SD; 95% CI, 0.03–0.15; *P* = 0.005).

**Table.** Results From Linear Regression Between Steps and Rate of Macular GCIPL Thinning in PROGRESSA Cohort

Model	Beta Coefficient (95% CI)^*^	*P* **^†^**
Univariable model		
Daily step count	0.09 (0.05, 0.12)	**<** **0.001**
Second model		
Daily step count	0.08 (0.04, 0.13)	**0.001**
Age	−0.006 (−0.012, −0.001)	**0.028**
Gender	−0.08 (−0.15, 0.04)	0.284
Final model		
Daily step count	0.07 (0.03,0.13)	**0.003**
Age	−0.007 (−0.01, −0.001)	**0.024**
Gender	−0.06 (−0.15, 0.033)	0.183
Highest IOP	−0.014 (−0.02, −0.006)	**<** **0.001**
Baseline average macular GCIPL thickness	0.003 (−0.002, 0.008)	0.271
Systolic blood pressure	−0.001 (−0.004, 0.001)	0.073
Diabetes mellitus	0.05 (−0.08, 0.18)	0.448

* Beta coefficient and 95% CIs represent the estimated effect of a covariate on macular thickness in micrometers. All continuous variables were scaled to internal *z*-scores centered on the study population mean prior to model fitting. Units for beta coefficients were µm/y/SD change for continuous variables or µm/y difference between outcomes for dichotomous variables.

† *P* values were derived from linear regression analyses. Boldface indicates *P* < 0.05.

This study then evaluated the association between the rate of macular GCIPL thinning and other metrics of physical activity (active calories and minutes spent doing moderate/vigorous activity). Both time spent doing moderate/vigorous activity and mean daily active calories were positively correlated with the rate of macular GCIPL thinning (moderate/vigorous activity: beta = (0.06 µm/y/SD; 95% CI, 0.01–0.105; *P* = 0.018; active calories: beta = 0.06 µm/y/SD; 95% CI, 0.006–0.114; *P* = 0.032). Secondary analysis evaluated associations with rate of peripapillary RNFL thinning. Daily step count was not correlated with the rate of peripapillary RNFL thinning (beta = 0.13 µm/y/SD; 95% CI, −0.02 to 0.28; *P* = 0.092).

### Association of Physical Activity and Macular Thickness in a Normative Population Cohort

This study then assessed the association between physical activity and retinal macular thickness in the UK Biobank. From a total sample of 502,448 participants, 96,679 had successfully completed the physical activity study with quality accelerometry data. From the potential 193,358 eyes in this sample, macular thickness data were available on 11,051 eyes. A further 2189 eyes (*n* = 1,547 participants) were excluded due to unavailable demographic and comorbidity data (all eyes had available ophthalmic data), leaving 8862 eyes from 6152 participants of the UK Biobank available for analysis (Fig. 1B). The mean age for this cohort was 54.9 ± 8.2 years, and 56.4% were female (Supplementary Table S2). The mean total macular thickness was 279 ± 12 µm, and the median physical activity was 27.8 milligravity (IQR, 23.4–33.4). Preliminary analysis of covariates determined that age, sex, IOP, eye, and hypertension were associated with macular thickness. Diabetes (*P* = 0.228), stroke (*P* = 0.595), and Alzheimer's disease (*P* = 0.197) were not associated with total macular thickness.

Physical activity illustrated a positive correlation with macular thickness in univariable analysis (beta = 1.1 µm/SD; 95% CI, 0.90–1.45; *P* < 0.001), in analysis accounting for age and gender (beta = 0.8 µm/SD; 95% CI, 0.53–1.13; *P* < 0.001), and in multivariable regression analysis (beta = 0.8 µm/SD; 95% CI, 0.47–1.14; *P* < 0.001) (Fig. 2B). These associations persisted in a subanalysis of 5885 participants with no history of ophthalmic disease (multivariable beta = 0.79 µm/SD; 95% CI, 0.45–1.12; *P* < 0.001).

## Discussion

This study investigated the association between physical activity with macular thickness in two cohorts. We initially demonstrated a positive association between physical activity and rates of longitudinal macular GCIPL thinning in 735 eyes from the PROGRESSA study. We then further evaluated this finding at a populational level by associated physical activity with total macular thickness in the UK Biobank.

Exercise has been proposed to correlate with rates of progression of various ophthalmic diseases. Lee and colleagues^9^ demonstrated correlations between physical activity and longitudinal visual field progression among 141 suspect and manifest glaucoma cases. Furthermore, a systematic review by McGuiness et al.^7^^,^^9^ conferred a lower risk of late age-related macular degeneration. Previous studies by Chrysostomou et al.^13^^,^^24^^,^^25^ using animal models have shown that physical activity promotes maintenance of a brain-derived neurotrophic factor that enables retinal ganglion cells to withstand cellular injury and degeneration. This study observed potential neuroprotective benefits of exercise among normative and glaucomatous cohorts. The observed correlation between activity and macular thickness in participants of the UK Biobank with no history of ophthalmic disease suggests that these findings may apply to normative healthy individuals. Furthermore, the observed correlation between longitudinal macular GCIPL thinning and accelerometer measured activity in the PROGRESSA implies that physical activity may be beneficial in longitudinal glaucomatous outcomes.

A major strength of this study was the use of accelerometer-acquired physical activity data. Our study used the Fitbit Inspire 2 and the Axivity AX3 devices to enable the acquisition of objective and reproducible physical activity data. The objective nature of these devices helped circumvent the self-reporting biases often observed in physical activity data acquired from questionnaires.^22^^,^^26^^–^^30^ Both devices are commercially available with open-source data processing, which enables reproducible metrics for physical activity.^22^^,^^31^^–^^34^ Furthermore, only one participant in the UK Biobank and only 13 participants did not finish the 7-day study period, suggesting a high ease of use. Finally, the 7-day study period aided in the acquisition of variations in physical activity between weekdays and weekends.

A key limitation is the use of cross-sectional data for physical activity. The singular cross-sectional 7-day study periods for physical activity limit the interpretation of these findings. Participants in the UK Biobank underwent physical activity measurement approximately 3 to 4 years after undergoing OCT imaging. Physical activity in the PROGRESSA study was retrospectively correlated with longitudinal SD-OCT data in the PROGRESSA cohort, thus similarly precluding the ability to assess whether activity predicts future progression. These limitations impede the ability to draw direct correlations between specific quantities of physical activity and rates of longitudinal macular thinning. In addition, it is conceivable that participants in both cohorts who are normally sedentary could have increased their step count during the 7-day period of study, and all participants who are normally active could have abruptly become more sedentary. This seems unlikely, however. Previous longitudinal studies have shown strong correlation between exercise measurements taken at different time points in a person's lifespan.^35^^–^^39^ Although physical activity does decline throughout life, these studies suggest that the most active people continue to be the most active for their age.^36^^,^^37^^,^^39^ Further longitudinal studies would be required to expand upon these findings to derive specific correlations between quantities and intensities of exercise and rates of retinal thinning and to delineate the effect of maintaining physical activity.

The collinearity among physical activity, ocular parameters, and systemic disease continues to be a limitation of observational studies.^19^^,^^40^^–^^44^ The correlations between physical activity and macular thinning may be in part due to confounding effects of age and the confounding and/or mediating effects of cardiovascular disease and/or IOP.^19^^,^^45^^,^^46^ For example, exercise has been demonstrated to reduce IOP, which may in part explain the observed associations in this study.^45^^,^^46^ This study sought to address this limitation by undertaking a combination of univariable and multivariable modeling that adjusted for demographic, ocular, and systemic predictors of macular thinning to enable the interpretation of results before and after adjustment for these parameters. It also depicted univariable analyses between physical activity and covariates to aid the evaluation of potential mediating pathways. Formally controlling for these parameters is unfortunately beyond the observational scope of this study. The results of this study may also be biased by the negative effects of vision impairment on physical activity.^4^^,^^9^^,^^25^ Given that physical activity in the PROGRESSA study was correlated with retrospective rates of macular thinning, it is plausible that the findings of this study are reflective of the deleterious effects of macular thinning on vision. This study sought to circumvent this limitation by demonstrating associations in subanalyses of participants characterized as glaucoma suspects and in participants of the UK Biobank without a history of ophthalmic disease. We ultimately recognize that to delineate the association between exercise and macular thickness may require an interventional study.^25^

The cohorts in this study represent a number of limitations. The UK Biobank is a randomly sampled population-based study of participants 40 to 70 years of age. This age group may have conferred the relatively low rate of neurodegenerative disorders in this group. Conversely, the PROGRESSA study included participants with features suspicious of or diagnostic of glaucoma, which may have introduced population biases, and the association between visual field deterioration and mobility introduces an inherent limitation.^47^^–^^50^ For this reason, this study demonstrated correlations between macular thinning and physical activity in cases without reproducible visual field loss. The use of multiple cohorts, however, did enhance the reproducibility of this work. Another limitation is the use of total macular thickness in the UK Biobank cohort. The results of this cohort may have been strengthened by the inclusion of macular GCIPL thickness data, which has better specificity for diseases such as glaucoma, but this unfortunately was not available from the UK Biobank at the time of analysis. This study also did not adjust for the ocular parameters. Ocular magnification has been demonstrated to confound measurement of SD-OCT–derived metrics.^1^ Furthermore, axial length has been correlated with both physical activity and macular GCIPL thickness, raising the possibility that these findings reflect an intercorrelation among myopia, physical activity, and macular thickness.^51^^–^^53^

Finally, it was beyond the scope of this study to formally assess various intensities of exercise.^11^ Ideally, this study would have incorporated exercise intensity data derived from a person's maximal oxygen uptake (VO_2max_).^54^^,^^55^ Given the burdensome nature of this, it was not feasible to perform such analyses in either cohort. Further evaluation of the neuroprotective effects of exercise would also require an interventional study design, such as a randomized cross-over design. The findings from this study may consequently pave the way for further research in this area.

This study identified a reproducible correlation between physical exercise and macular thinning. Greater physical activity was associated with slower macular GCIPL thinning in the PROGRESSA study, highlighting a potential neuroprotective role for physical activity. Further work utilizing interventional study designs is required to formally investigate the influence of exercise in ophthalmic diseases.

## Supplementary Material

Supplement 1
