# Supplementary material for: Physical Activity Is Associated With Macular Thickness: A Multi-Cohort Observational Study
Source: Invest Ophthalmol Vis Sci. 2023 Mar 3;64(3):11. doi: 10.1167/iovs.64.3.11 (PMC9988706; doi:10.1167/iovs.64.3.11)
Supplement: Supplement 1 [file iovs-64-3-11_s001.pdf]

SUPPLEMENTARY RESOURCES

Supplementary Table 1: Summary Statistics for PROGRESSA Cohort

	PROGRESSA Summary Statistics (n = 735 eyes / 388 participants)
**Demographic Characteristics**	
**Age (years)**	70.4±8.8
**Sex**	
**Female**	420 / 220 [56.7]
**Male**	315 / 168 [43.3]
**Socio-economic Decile**	6.4±2.8
**Cardiovascular Characteristics**	
**Systolic Blood Pressure (mmHg)**	137.2±20.4
**Diabetes**	
**Yes**	107 / 55 [14.2]
**No**	626 / 333 [85.8]
**Ocular Characteristics**	
**Topical Glaucoma Therapy**	
**Yes**	322 / 170 [43.8]
**No**	413 / 218 [52.2]
**Highest Intraocular Pressure (mmHg)**	20.6±5.5
**Glaucoma Characterization**	
**Early Manifest Glaucoma**	264 / 142 [36.5]
**Suspect**	471 / 246 [63.5]
**Baseline average macular GCIPL thickness (um)**	74.9±8.2

Supplementary Table 1 summarizes the baseline demographic, cardiovascular and ocular characteristics for PROGRESSA study participants. Summary statistics for continuous variables are reflected as mean±standard deviation, and summary statistics for discrete variables are reflected as number (n) of eyes followed by number of participants (n of eyes / n of participants) with proportion of participants for each characteristic displayed in parenthesis. Age and socio-economic decile are represented as per participant summary. Highest intraocular pressure and baseline macular GCIPL thickness are displayed as per eye summary.

Supplementary Table 2: UK Biobank Summary Statistics

	UK Biobank Summary Statistics (n = 8862 eyes / 6152 participants)
**Demographic Characteristics**	
**Age (years)**	54.9±8.2
**Sex**	
**Female**	4985 / 3471 [56.4]
**Male**	3877 /2681 [43.6]
**Systemic Characteristics**	
**Hypertension**	
**Yes**	2062 / 1462 [23.7]
**No**	6800 / 4690 [76.7
**Diabetes**	
**Yes**	263 / 180 [2.9]
**No**	8599 / 5972 [97.1]
**Stroke**	
**Yes**	70 / 52 [0.8]
**No**	8792 / 6100 [99.2]
**Alzheimers Disease (n [%])**	
**Yes**	30 / 23 [0.4]
**No**	8832 / 6129 [99.6]
**IOP (mmHg)**	15.3±3.1

Supplementary Table 2 summarizes the baseline demographic, cardiovascular and ocular characteristics for UK Biobank study participants. Summary statistics for continuous variables are reflected as mean±standard deviation, and summary statistics for discrete variables are reflected as number (n) of eyes followed by number of participants (n eyes / n participants) with proportion of participants for each characteristic displayed in parenthesis. Age is represented as per participant summary. Intraocular pressure is displayed as per eye mean intraocular pressure.
